# Supplementary material for: Drug repurposing for aging research using model organisms
Source: Aging Cell. 2017 Jun 16;16(5):1006–15. doi: 10.1111/acel.12626 (PMC5595691; doi:10.1111/acel.12626)
Supplement: Supplementary file 7 — Data S1 Zip‐Archive of all report cards. [file ACEL-16-1006-s007.zip › RC_NIL.pdf]

NIL

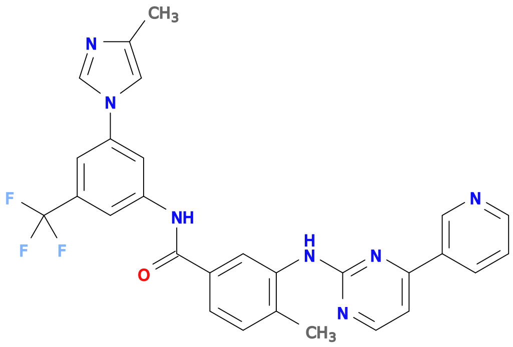

Database identifiers

ChEMBLCompound	CHEMBL255863
DrugBank	DB04868
CHEBI	52172
ZINC	ZINC06716957
eMolecules	10645209

Ranking

	Rank	Score
Drosophila	15/697	0.91
C. elegans	2/591	0.909

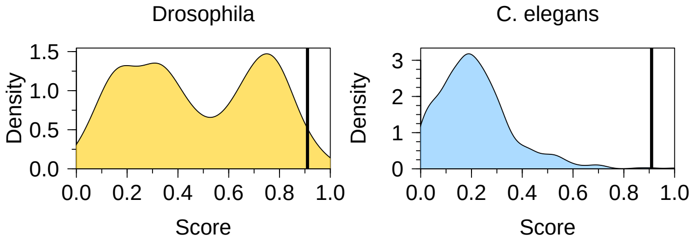

	Ageing implication	Domain conservation	Binding site conservation	Binding affinity	Bioavailability	Lipinski	Promiscuity	Purchasability	Drug approval	**Total**
Drosophila	1.0	0.966	1.0	0.933	(0.9)	-0.1	-0.001	0.1	0.1	0.91
C. elegans	1.0	0.952	0.956	0.933	0.954	-0.1	-0.001	0.1	0.1	0.909

Names

- **nilotinib**
- Amn 107
- Tasigna

Roles

antineoplastic agent, tyrosine kinase inhibitor

Status

Approved drug (according to ChEMBL)	Yes
Number of Rule of 5 violations	2
Binding affinity to original target in log units (RF-Score prediction)	7.64
Burns *C. elegans* bioavailability prediction	10.31

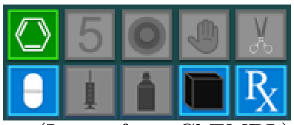

(Image from ChEMBL)

Medical Information

Indicatation: For the potential treatment of various leukemias, including chronic myeloid leukemia (CML).
Mechanism of action: Chronic myelogenous leukaemia (CML) is caused by the BCR-ABL oncogene. Nilotinib inhibits the tyrosine kinase activity of the BCR-ABL protein. Nilotinib fits into the ATP-binding site of the BCR-ABL protein with higher affinity than imatinib, over-riding resistance caused by mutations. The ability of AMN107 to inhibit TEL-platelet-derived growth factor receptor-beta (TEL-PDGFRbeta), which causes chronic myelomonocytic leukaemia, and FIP1-like-1-PDGFRalpha, which causes hypereosinophilic syndrome, suggests potential use of AMN107 for myeloproliferative diseases characterised by these kinase fusions (Stover et al, 2005; Weisberg et al, 2005). AMN107 also inhibits the c-Kit receptor kinase, including the D816V-mutated variant of KIT, at pharmacologically achievable concentrations, supporting potential utility in the treatment of mastocytosis, and gastrointestinal stromal tumours (Weisberg et al, 2005; von Bubnoff et al, 2005; Gleixner et al, 2006).
Retail: Novartis pharmaceuticals corp

(Information from DrugBank)

Compound Target Characteristics

Tyrosine-protein kinase ABL1

Best gene implication in ageing for this target family came from gene P00519 via mapping the annotation from Ensembl ENSG00000097007 via mapping the annotation from EntrezGene 25 via mapping the annotation from GenAgeHuman 0078 annotated in GenAge release 17.

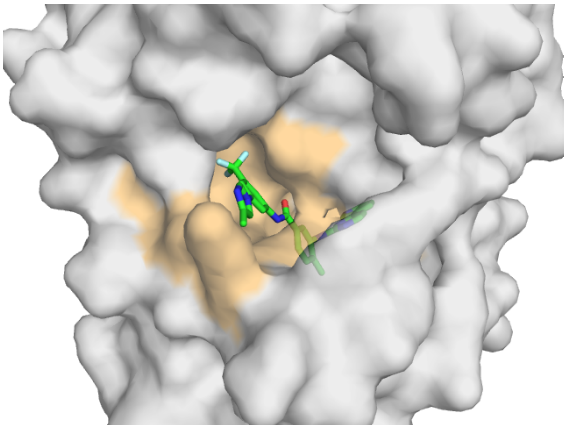

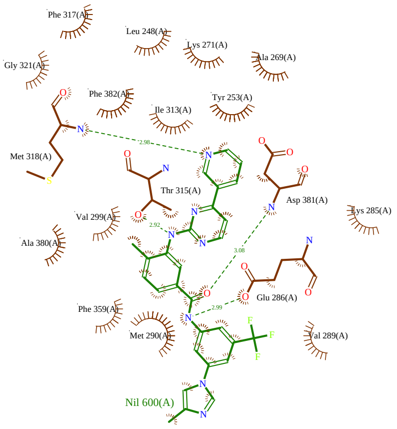

protein	amino acids contacts (binding site)
PDB:3cs9:chainA:P00519	L Y A K K E V M I L V I T F M G F H V A D F
PDB:3cs9:chainB:P00519	L Y A K K E V M I L V I T F M G F H V A D F
sp:P00519:ABL1_HUMAN	L Y A K K E V M I L V I T F M G F H V A D F
tr:Q59FK4:Q59FK4_HUMAN	L Y A K K E V M I L V I T F M G F H V A D F
tr:F1M0A6:F1M0A6_RAT	L Y A K K E V M I L V I T F M G F H V A D F
tr:D3ZGM3:D3ZGM3_RAT	L Y A K K E V M I L V I T F M G F H V A D F
tr:E9PT20:E9PT20_RAT	L Y A K K E V M I L V I T F M G F H V A D F
tr:Q3V2W1:Q3V2W1_MOUSE	L Y A K K E V M I L V I T F M G F H V A D F
sp:P00520:ABL1_MOUSE	L Y A K K E V M I L V I T F M G F H V A D F
tr:Q3SYK5:Q3SYK5_MOUSE	L Y A K K E V M I L V I T F M G F H V A D F
sp:P00522:ABL_DROME	L Y A K E E I M M L V I T F M G Y H V A D F
tr:A8JNU2:A8JNU2_DROME	L Y A K E E I M M L V I T F M G Y H V A D F
sp:P03949:ABL1_CAEEL	L Y A K A E I M L L V I T F M G F H I A D F

protein	whole protein ident	simil	domain-based ident	simil	contact-based ident	simil
PDB:3cs9:chainA:P00519	1.0	1.0	1.0	1.0	1.0	1.0
PDB:3cs9:chainB:P00519	1.0	1.0	1.0	1.0	1.0	1.0
sp:P00519:ABL1_HUMAN	1.0	1.0	1.0	1.0	1.0	1.0
tr:Q59FK4:Q59FK4_HUMAN	0.95	0.96	1.0	1.0	1.0	1.0
tr:F1M0A6:F1M0A6_RAT	0.84	0.93	1.0	1.0	1.0	1.0
tr:D3ZGM3:D3ZGM3_RAT	0.87	0.95	1.0	1.0	1.0	1.0
tr:E9PT20:E9PT20_RAT	0.84	0.93	1.0	1.0	1.0	1.0
tr:Q3V2W1:Q3V2W1_MOUSE	0.3	0.32	1.0	1.0	1.0	1.0
sp:P00520:ABL1_MOUSE	0.87	0.95	1.0	1.0	1.0	1.0
tr:Q3SYK5:Q3SYK5_MOUSE	0.84	0.93	1.0	1.0	1.0	1.0
sp:P00522:ABL_DROME	0.26	0.49	0.78	0.93	0.82	0.95
tr:A8JNU2:A8JNU2_DROME	0.25	0.48	0.78	0.93	0.82	0.95
sp:P03949:ABL1_CAEEL	0.31	0.62	0.67	0.9	0.82	0.93

Abl (FBgn0000017) associated phenotypes

cell migration defective, cell shape defective, germline clone, heat sensitive, lethal - all die before end of pupal stage, maternal effect, mitotic cell cycle defective, neuroanatomy defective, neurophysiology defective, partially lethal - majority die, partially lethal - majority live, reduced, rescuable maternal effect, size defective, somatic clone, some die during pupal stage

(Information from FlyBase)

Abl (UniProt:P00522) annotation

Function: Arm and Abl proteins function cooperatively at adherens junctions in both the CNS and epidermis; critical for embryonic epithelial morphogenesis regulating cell shape changes and cell migration. Plays a critical role in transducing embryonic midline repulsive cues; may regulate cytoskeletal dynamics underlying a growth cone's response to midline cues. The ability of pCC/MP2 axons to correctly interpret midline repulsive cues and stay on the ipsilateral side is dependent on the strength of both Slit/robo and Abl-dependent signaling pathways. (PubMed:11756472, PubMed:12973825, PubMed:9635189).

Subcellular location: Cytoplasm.

Tissue specificity: Arm and ena colocalize with Abl at adherens junctions throughout development. (PubMed:11756472, PubMed:9635189).

Developmental stage: Expressed both maternally and zygotically. (PubMed:2832740).

Disruption phenotype: Both loss- and gain-of-function mutants exhibit neurons within the pCC/MP2 pathway that incorrectly project across the midline. Loss of Abl disrupts cell migration and cell shape changes during dorsal closure. (PubMed:11756472, PubMed:9635189).

(Information from UniProt)

abl-1 (WBGene00000018) associated phenotypes

apoptosis variant, gamma ray induced apoptosis increased, lipid synthesis increased, protein subcellular localization variant

(Information from WormBase)

Mitogen-activated protein kinase 11

Best gene implication in ageing for this target family came from gene O62618 annotated in UniProt release 2014_02. Annotation GO 8340 (determination of adult lifespan) was Inferred from Mutant Phenotype

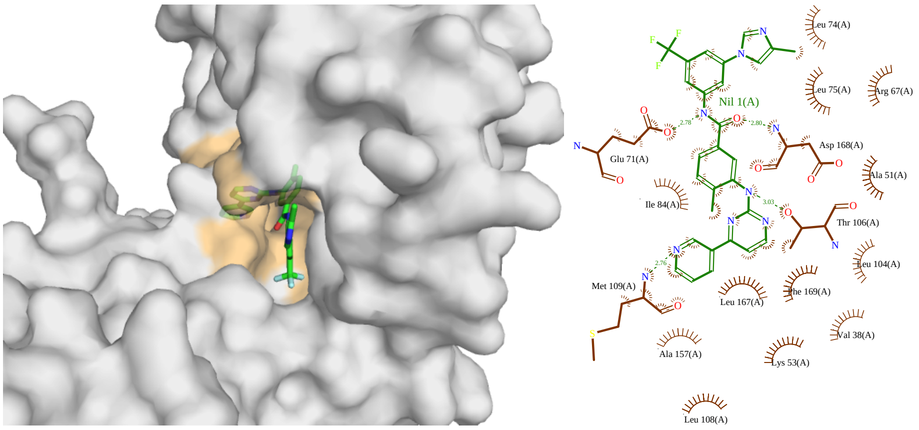

protein	amino acids contacts (binding site)																
PDB:3gp0:chainA:Q15759	V	A	K	R	E	L	L	V	I	L	T	L	M	H	A	L	D	F
sp:Q15759:MK11_HUMAN	V	A	K	R	E	L	L	V	I	L	T	L	M	H	A	L	D	F
tr:L7RSM2:L7RSM2_HUMAN	V	A	K	R	E	L	L	V	I	L	T	L	M	H	A	L	D	F
sp:Q16539:MK14_HUMAN	V	A	K	R	E	L	L	V	I	L	T	L	M	H	A	L	D	F
tr:D4A3U7:D4A3U7_RAT	V	A	K	R	E	L	L	V	I	L	T	L	M	H	A	L	D	F
tr:G3V617:G3V617_RAT	V	A	K	R	E	L	L	V	I	L	T	L	M	H	A	L	D	F
tr:Q56A33:Q56A33_RAT	V	A	K	R	E	L	L	V	I	L	T	L	M	H	A	L	D	F
tr:B2KF34:B2KF34_MOUSE	V	A	K	R	E	L	L	V	I	L	T	L	M	H	A	L	D	F
tr:B2KF35:B2KF35_MOUSE	V	A	K	R	E	L	L	V	I	L	T	L	M	H	A	L	D	F
sp:P47811:MK14_MOUSE	V	A	K	R	E	L	L	V	I	L	T	L	M	H	A	L	D	F
sp:Q9WUI1:MK11_MOUSE	V	A	K	R	E	L	L	V	I	L	T	L	M	H	A	L	D	F
tr:Q5U421:Q5U421_MOUSE	V	A	K	R	E	L	L	V	I	L	T	L	M	H	A	L	D	F
sp:O62618:MK14A_DROME	V	A	K	R	E	L	L	V	I	L	T	L	M	H	A	L	D	F
tr:E1JIV6:E1JIV6_DROME	V	A	K	R	E	L	L	V	I	L	T	L	M	H	A	L	D	F
sp:Q17446:PMK1_CAEEL	V	A	K	R	E	L	L	I	I	F	S	L	M	H	A	L	D	F
sp:Q8MXI4:PMK2_CAEEL	V	A	K	R	E	L	L	V	L	F	S	L	M	H	A	L	D	F
sp:P32485:HOG1_YEAST	V	A	K	R	E	L	L	L	I	F	T	L	Q	H	L	C	D	F

protein	whole protein ident	whole protein simil	domain-based ident	domain-based simil	contact-based ident	contact-based simil
PDB:3gp0:chainA:Q15759	1.0	1.0	1.0	1.0	1.0	1.0
sp:Q15759:MK11_HUMAN	1.0	1.0	1.0	1.0	1.0	1.0
tr:L7RSM2:L7RSM2_HUMAN	0.71	0.92	0.75	0.94	1.0	1.0
sp:Q16539:MK14_HUMAN	0.73	0.92	0.78	0.95	1.0	1.0
tr:D4A3U7:D4A3U7_RAT	0.97	0.99	0.99	1.0	1.0	1.0
tr:G3V617:G3V617_RAT	0.74	0.92	0.78	0.95	1.0	1.0
tr:Q56A33:Q56A33_RAT	0.71	0.92	0.75	0.94	1.0	1.0
tr:B2KF34:B2KF34_MOUSE	0.46	0.53	0.52	0.6	1.0	1.0
tr:B2KF35:B2KF35_MOUSE	0.56	0.67	0.65	0.77	1.0	1.0
sp:P47811:MK14_MOUSE	0.73	0.92	0.78	0.95	1.0	1.0
sp:Q9WUI1:MK11_MOUSE	0.97	0.99	0.99	1.0	1.0	1.0
tr:Q5U421:Q5U421_MOUSE	0.71	0.92	0.75	0.94	1.0	1.0
sp:O62618:MK14A_DROME	0.61	0.85	0.69	0.9	1.0	1.0
tr:E1JIV6:E1JIV6_DROME	0.61	0.85	0.69	0.9	1.0	1.0
sp:Q17446:PMK1_CAEEL	0.59	0.85	0.68	0.9	0.83	0.94
sp:Q8MXI4:PMK2_CAEEL	0.47	0.74	0.57	0.83	0.83	0.96
sp:P32485:HOG1_YEAST	0.38	0.68	0.51	0.84	0.72	0.77

Mpk2 (FBgn0015765) associated phenotypes

heat stress response defective, immune response defective, non-enhancer of variegation, non-suppressor of variegation, nutrition conditional, osmotic stress response defective, oxidative stress response defective, partially lethal - majority die, reduced, short lived, starvation stress response defective, stress response defective

(Information from FlyBase)

Mpk2 (UniProt:O62618) annotation
Function: Kinase involved in a signal transduction pathway. May down-regulate insect immunity gene expression after prolonged infection. (PubMed:9417090, PubMed:9584193).
Cofactor:Mg(2+)
Enzyme regulation: Activated by threonine and tyrosine phosphorylation by Mkk3 in response to environmental stress. (PubMed:9417090, PubMed:9584193).
Subcellular location: Nucleus (PubMed:9417090).
Developmental stage: Expressed both maternally and zygotically. Levels are highest at the preblastoderm stage but low levels are present throughout development. (PubMed:9584193).
Domain: The TXY motif contains the threonine and tyrosine residues whose phosphorylation activates the MAP kinases.
Ptm: Dually phosphorylated on Thr-184 and Tyr-186, which activates the enzyme. (PubMed:9417090).
(Information from UniProt)

pmk-1 (WBGene00004055) associated phenotypes
cell stress response variant, drug induced gene expression variant, gene expression level reduced, lethal, life span variant, organism oxidative stress response hypersensitive, pathogen induced gene expression variant, pathogen susceptibility increased, sterile
(Information from WormBase)

pmk-1 (UniProt:Q17446) annotation
Function: Responds to activation by environmental stress and pro- inflammatory cytokines by phosphorylating downstream targets. Functions downstream of the MAPKK sek-1 and the MAPKKK nsy-1 as the MAP kinase required for pathogen resistance. (PubMed:11703092, PubMed:12142542).
Cofactor: Mg(2+) Evidence=(PubMed:11703092);
Enzyme regulation: Activated by phosphorylation on threonine and tyrosine. Inhibited by pyridinyl-imidazole related compounds. (PubMed:11703092).
Domain: The TXY motif contains the threonine and tyrosine residues whose phosphorylation activates the MAP kinases.
Ptm: Dually phosphorylated on Thr-191 and Tyr-193, which activates the enzyme.
(Information from UniProt)

pmk-2 (UniProt:Q8MXI4) annotation
Function: Responds to activation by environmental stress and pro- inflammatory cytokines by phosphorylating downstream targets. (PubMed:11703092).
Cofactor: Mg(2+) Evidence=(PubMed:11703092);
Enzyme regulation: Activated by phosphorylation on threonine and tyrosine. Inhibited by pyridinyl-imidazole related compounds. (PubMed:11703092).
Subcellular location: Cytoplasm (PubMed:11703092).
Domain: The TXY motif contains the threonine and tyrosine residues whose phosphorylation activates the MAP kinases.
Ptm: Dually phosphorylated on Thr-222 and Tyr-224, which activates the enzyme.
(Information from UniProt)
